# Intersecting transcriptomic profiling technologies and long non-coding RNA function in lung adenocarcinoma: discovery, mechanisms, and therapeutic applications

**DOI:** 10.18632/oncotarget.18432

**Published:** 2017-06-09

**Authors:** Jonathan Castillo, Theresa R. Stueve, Crystal N. Marconett

**Affiliations:** ^1^ Department of Surgery, Keck School of Medicine, University of Southern California, Los Angeles, CA, USA; ^2^ Department of Biochemistry and Molecular Medicine, Keck School of Medicine, University of Southern California, Los Angeles, CA, USA; ^3^ Department of Norris Comprehensive Cancer Center, Keck School of Medicine, University of Southern California, Los Angeles, CA, USA

**Keywords:** LncRNA biology, transcriptomic analysis, lung adenocarcinoma, cancer, RNA biology

## Abstract

Previously thought of as junk transcripts and pseudogene remnants, long non-coding RNAs (lncRNAs) have come into their own over the last decade as an essential component of cellular activity, regulating a plethora of functions within multicellular organisms. lncRNAs are now known to participate in development, cellular homeostasis, immunological processes, and the development of disease. With the advent of next generation sequencing technology, hundreds of thousands of lncRNAs have been identified. However, movement beyond mere discovery to the understanding of molecular processes has been stymied by the complicated genomic structure, tissue-restricted expression, and diverse regulatory roles lncRNAs play. In this review, we will focus on lncRNAs involved in lung cancer, the most common cause of cancer-related death in the United States and worldwide. We will summarize their various methods of discovery, provide consensus rankings of deregulated lncRNAs in lung cancer, and describe in detail the limited functional analysis that has been undertaken so far.

## RECOGNITION FOR THE DIVERSITY OF LNCRNAS AND THEIR INVOLVEMENT IN CANCER

The first lncRNA was discovered decades ago during the characterization of X-chromosome inactivation [1]. Following that early discovery, several lncRNAs were inadvertently uncovered and characterized as anomalous molecules before the community recognized that lncRNAs represent a distinct class of regulatory RNAs. With the completion of the human genome project in 2003 and subsequent characterization the genomic landscape, attempts at bioinformatics prediction of mRNA genes was found to be cluttered with many fold higher predicted transcripts than were experimentally verified as the precursors of proteins [2–5]. What these programs revealed was a glut of predicted transcripts, genes with hallmarks of transcription but no discernable protein coding function. In addition, these were thought to have no practical biological function because they had little evolutionary conservation [6]. Initially, these unverified genes were dismissed as programming artifacts to be eliminated [7–9]. However, it was quickly realized the lack of evolutionary conservation did not rule out function [10]. It is now accepted that the human genome contains many thousands of lncRNA transcripts. Functional implications of this discovery have yet to be fully elucidated. To date lncRNAs have been detected throughout development and in every cell type tested thus far.

**Figure 1:**
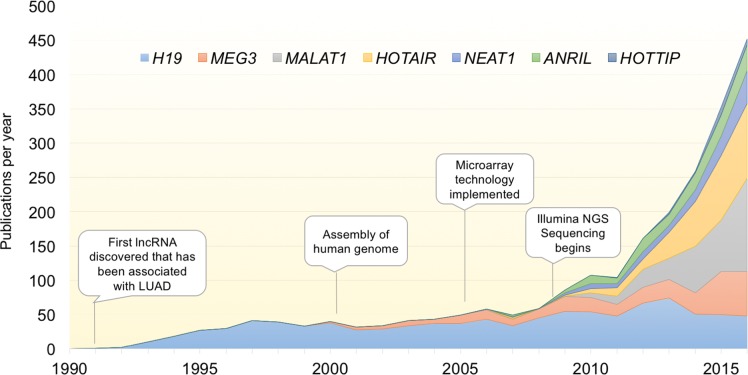
Exponential discovery of lncRNAs with the adoption of transcriptome-wide gene expression technologies Graph indicates the total number of publications per year for select lncRNAs with known involvement in LUAD. With the advent of transcriptomic profiling, the pace of lncRNA discovery and papers characterizing their function has increased exponentially over the last decade. lncRNAs included are those described in Table 1.

One field that has been particularly active in lncRNA discovery is cancer biology. Due to the pressing need for development of novel therapeutics and diagnostics, many newly emergent fields have been focused on cancer research. These include the discovery of microRNAs, targeted immunotherapy, and most recently circulating tumor cells. Added to this ever-growing list are lncRNAs. Their implication in a diverse array of regulatory roles has heightened interest in these molecules as functional players in the development and heterogeneity of cancer [11]. Recently, the pace of discovery and functional validation for lncRNAs has been increasing exponentially with the advent of sequencing technologies (Figure 1). But due to the rapid pace of discovery little headway has been made in functionally characterizing the bulk of these newly-discovered genes.

**Figure 2:**
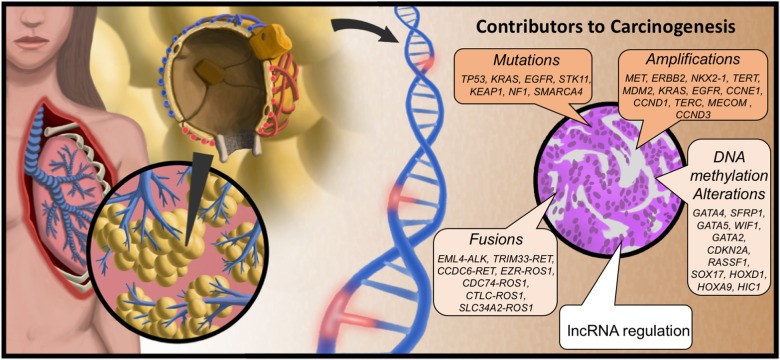
Molecular origins of LUAD Lung adenocarcinoma (LUAD) arises in the distal alveolar epithelium from progenitor alveolar epithelial cells. LUAD develops from these precursor cells though oncogenic activation (and deactivation of tumor suppressors) by induced mutations to the DNA, amplification and fusion events, as well as epigenomic alterations. Genes listed were taken from TCGA analysis of LUAD (15). Added to this is the newly-emergent appreciation for altered lncRNA regulation of cellular processes as an oncogenic event.

One cancer type with particularly high mortality is lung cancer. The overall mortality rate (all stages) remains at ∼85% [12], which is comparable to stage IV breast cancer. The landmark National Lung cancer Screening Trial (NLST) study identified Spiral CT as an effective detection tool that reduced overall mortality, however the study was only applicable to lifelong smokers and has a false positive rate of 92% [13]. One issue complicating the development of early detection strategies is that lung cancer is composed of several distinct subtypes, each with their own etiology and clinical outcomes. Lung cancer is loosely broken into two subcategories, small cell (SCLC, ∼13% of cases) and non-small cell lung cancer (NSCLC, ∼87% of cases). Lung adenocarcinoma (LUAD) is the most common subtype of NSCLC, and arises from the distal alveolar epithelium (Figure 2). This cancer has been linked to mutations in *EGFR* for never-smokers and mutations in *kRAS* for smokers [14]. In addition, dozens of other oncogenic mutations, copy number variations, and epigenetic alterations have been described in LUAD [15, 16]. Several oncogenic mutations in protein coding genes have been exploited for the development of targeted therapeutics. Notably among them are Erlotinib and Gefintinib, both EGFR inhibitors, and Crizotinib, an ALK/ROS1/MET kinase inhibitor [17–20]. While Erlotinib and Gefitinib are in use clinically, each is associated with a high rate of relapse in patients due to further molecular alterations that develop, such as the 790M mutation to EGFR, which renders the cancer resistant [21]. Therefore, there is a pressing need to both define molecular hallmarks that distinguish LUAD from other lung cancers and normal tissues, and to specifically target those cancerous cells while leaving lung function intact.

In this review, we focus on lncRNAs with characteristics indicating they could be exploited in improved efficacy of LUAD detection, clinical management, and outcome prediction. We first outline the current state of molecular characterization for lncRNAs with known involvement in LUAD etiology. Then, we utilize multiple high-throughput analysis recently made publicly available to define a subset of high-interest candidate lncRNAs. Of these, we provide a synopsis on what is currently known about the predicted candidates. We end with discussion of ways in which knowledge of dysregulated lncRNAs in LUAD can be leveraged in the clinic.

## KNOWN LNCRNAS INVOLVED IN LUAD

The biological significance of lncRNAs is under intense investigation. Because lncRNAs were grouped into a broad category of any non-coding RNA longer than 200 nucleotides, this class of RNAs represents a heterogeneous group in terms of mechanism and function. lncRNAs are implicated in transcriptional regulation, cellular signaling, chromatin remodeling, splicing, and a host of other processes [22–25]. Mechanistically, lncRNAs can regulate transcriptional activity at the endogenous locus through antisense activity and *in trans* through the regulation of epigenetic structure [26, 27]. At the post-transcriptional level, lncRNAs regulate splicing, micro-RNA targeting, and through RNA-protein interactions, can influence their binding partner function, localization, and activity [28–30]. In terms of biological processes, lncRNAs are involved in regulation of the cell cycle, apoptosis, differentiation, and immunological response [31–34]. Despite the large repertoire of lncRNAs expressed in lung, only a handful have been functionally linked to LUAD development. Some exhibit hallmarks of tumor suppression, such as *MEG3* [35], while others, such as *HOTAIR*, behave as oncogenes through increased proliferation and reduced survival [36]. Table 1 highlights some of the known lncRNAs involved in LUAD and their cellular mechanism of action. However, for most lncRNAs, a defined molecular mechanism has yet to be discovered.

**Table 1:** Known lncRNAs involved in LUAD

GENE	Locus	Nearby factor implicated in cancer	Mechanism(s) of action	Additional cancer association(s)
*ANRIL*	9p21	CDKN2B (INK4-ARF) tumor suppressor	Oncogene. Antagonizes the CDKN2A and CDKN2B tumor suppressors via recruitment of PRC2 and PRC1 [57,58].	basal cell, breast, cervical, esophageal, gallbladder, gastric, liver, melanoma, ovarian.
*H19*	11p15	IGF2 growth factor	Oncogene. Targets multiple tumor suppressive miRNAs [29,45]; parent transcript of miRNAs involved in regulation of tight junction dynamics [44,46].	adrenal, bladder, cervical, colorectal, gallbladder, gastric, esophageal, laryngeal, nasopharyngeal, ovarian, pancreatic, thyroid.
*HOTAIR*	12q13	HOXC transcription factors	Oncogene. Long-range epigenetic action via recruitment of PRC2 and LSD1 [65–67]; serves as a miRNA sponge to block miR-331-3p mediated destruction of HER2 transcripts [68].	bladder, colorectal, ER (+) breast, liver, nasopharyngeal, oral, ovarian, pancreatic, pituitary, small cell lung.
*HOTTIP*	7p15	HOXA transcription factors	Oncogene. Regulates chromatin structure at the HOXA transcription factor locus [98].	colorectal, pancreatic, osteosarcoma, tongue.
*MALAT1*	11q13	*NEAT1* lncRNA	Oncogene. Suppresses E-cadherin via suz12 recruitment, leading to metastasis [112].	glioma, multiple myeloma, pituitary, renal clear cell, tongue.
*MEG3*	14q32	DLK1 growth factor receptor	Tumor Suppressor. Long-range epigenetic action, leading to suppression of TFG β [90]; blocks oncogenic activity of miR-21 [86].	AML, cervical, colorectal, gastric, meningioma, ovarian, pancreatic, pituitary, prostate, thyroid.
*NEAT1*	11q13	*MALAT1* lncRNA	Oncogene. Promotes survival when DNA damage present via paraspeckle formation [122,123].	colorectal, esophageal, gastric, glioma, leukemia, ovarian, prostate.

The RefSeq gene name annotation, alongside the hg19 chromosomal location are listed. In addition, the established mechanism of action is listed, as well as other cancers where the lncRNA has demonstrated effects on tumor initiation, promotion, progression, and/or patient survival outcomes.

### Pan-cancer lncRNAs

It is important to note that, while the lncRNAs in Table 1 play a role in LUAD development, they are all implicated in the development of multiple cancer types, and therefore do not confer specificity to any given cancer. Because multiple types of cancers depend on similar pathways for sustained growth, it is not surprising that a subset of lncRNAs have been linked to suppression of p53, Wnt signaling activation, epithelial to mesenchymal transition (EMT), and similar early steps in the process of oncogenesis. Here, we discuss examples of lncRNAs that not only promote LUAD but are also involved in tumorigenesis in a variety of cancers.

#### H19

The maternally expressed and imprinted gene *H19* is elevated in numerous cancers. Overexpression occurs through the loss of epigenetic repression at the paternal allele [37–39]. More recently, *H19* was found to be upregulated in NSCLC tissue and correlated with poor prognosis [40]. Many genes involved in embryonic growth and implicated in cancer lie within the *H19* locus and are *cis* regulated by *H19* [41, 42]. In addition, *H19* upregulation has been linked to MYC oncogene activation [43]. Therefore, disrupted paternal imprinting on *H19* acts as an oncogenic driver in several cancers, including NSCLC.

Mechanistically, *H19* serves as the precursor miR-675, which is processed from the first exon of *H19* and in its mature miRNA form mediates degradation of ZO-1 and E-Cadherin mRNA, disrupting tight junction formation, which in turn disrupts epithelial architecture and leads to increased invasion [44, 45]. In addition to being a precursor to miRNAs, *H19* can suppress several miRNAs, including *let-7* [46], *miR-138,* and *miR-200a* [29] by serving as a competing endogenous RNA (ceRNA). Suppression of both *miR-138* and *miR-200a* via *H19* was shown to re-activate expression of the mesenchymal marker genes ZEB1, ZEB2, and Vimentin, resulting in EMT progression in bladder cancer [29]. In addition, *H19* was recently shown to suppress miR-141 and miR-22, both of which function as antagonists of Wnt signaling [47, 48]. This *H19*-mediated suppression lead to the activation of the Wnt/β-catenin pathway during osteoblast differentiation [49]. Their role in Wnt/B-catenin signaling might suggest an alternative means by which *H19* can promote tumorigenesis. In addition, *H19* has been linked with Wnt-mediated tumorigenesis via PRC2/EZH2 recruitment to the Wnt antagonist gene *NKD1* [50]. The convergent activity of *H19* on different parts of the Wnt signaling pathway is interesting from both a mechanistic and evolutionary standpoint, revealing that individual lncRNAs can perform multiple functional roles simultaneously to effect intracellular signaling cascades.

*H19* has also been shown to negatively regulate p53 signaling. Ectopic expression of *H19* can cause increased cell growth and decreased p53 transcriptional activity [51]. This was attributed to a physical interaction between *H19* and the p53 protein. However, the mechanism by which this interaction mediates the inactivation of p53 remains ambiguous and more investigation is needed to fully evaluate the effect of *H19* on p53-mediated cellular arrest and apoptosis.

#### ANRIL (antisense non-coding RNA in the INK4 locus)

This gene lies within the 9p21.3 gene cluster, consisting of the p14^ARF^, p15^INK4b^, and p16^INK4a^ tumor suppressor genes. Within this locus, *ANRIL* is the natural antisense transcript of the p16^INK4a^ gene. p14^ARF^ is involved in stabilizing p53 levels by negatively regulating MDM2 [52], whereas both p15^INK4b^ and p16^INK4a^ are critical regulators of the cell cycle [53]. Their deactivation promotes an increase in cellular proliferation and is seen in several cancers [54, 55]. In addition, the deactivation of the 9p21.3 gene cluster often occurs in conjunction with LUAD driven by mutationally-activated kRAS [56]. The proximity of *ANRIL* within the gene cluster allows for a *cis*-mediated suppression of p16^INK4a^ that occurs through recruitment of PRC2 complex which compacts chromatin and subsequently deactivates gene expression [57, 58]. A more recent study demonstrated that *ANRIL* is overexpressed in NSCLC, correlating with poor prognosis [59].

#### HOTAIR (HOX transcript antisense RNA)

This well-characterized lncRNA has been the sole subject of previous reviews [36, 60–63], and acts by binding and promoting chromatin compaction through association with GA-rich DNA sequence motifs that subsequently recruit PRC2 [64–66]. This results in genome-wide epigenetic regulation of differentiation and cancer development [67]. *HOTAIR* can also regulate miRNA by acting as a competing endogenous RNA (ceRNA) to deplete cells of miR-331-3p, enhancing expression of the HER2 receptor tyrosine kinase and thereby promoting oncogenesis [68]. Indeed, *HOTAIR* displays all the canonical behaviors of an oncogene, including poor prognosis when present [69, 70], chemoresistance [71], reduced overall survival [72–75], and increased metastasis [76–80]. This occurs in a number of cancers, including both SCLC [81] and NSCLC [82, 83].

#### MEG3 (maternally expressed gene 3)

One of many maternally imprinted lncRNAs [84], *MEG3* exhibits the hallmarks of a tumor suppressor, namely inhibition of proliferation and induction of apoptosis [35, 85–87] in numerous cancers. Multiple functions for *MEG3* in cancer have been described [87, 88], Locally, expression is inversely correlated with the nearby tumor suppressor *DLK1*, which it may regulate [89]. *MEG3* can also act throughout the genome as an epigenomic regulator of TGFβ-responsive distal regulatory elements. It does so by forming RNA:DNA triplex helix structures at GA-rich sequence recognition sites, which bring EZH2 to target loci, effectively condensing local chromatin regions to disrupt enhancer activity and block TGFβ-induced proliferation [90]. Additionally, *MEG3* can mediate the destruction of miR-21, blocking this microRNAs oncogenic potential [86].

It is interesting to note that both *HOTAIR* and *MEG3* bind GA-rich sequence elements that facilitate recruitment of PRC2 complex and condense the local chromatin environment, yet *HOTAIR* functions as an oncogene while *MEG3* functions as a tumor suppressor. Considering that *HOTAIR* oncogenic activity is seen in multiple cancers and *MEG3* tumor suppressor activity is also observed across cancers, the simple explanation of differing mechanisms in differing tumors does not seem applicable. Instead, follow up studies on the genomic distribution of the two lncRNAs, their relative expression to each other, and any mechanistic interactions they may have are warranted to address this question.

#### HOTTIP (HOXA transcript at the distal TIP)

*HOTTIP* is another lncRNA transcribed from the HOXA locus which exhibits the oncogenic properties of increased proliferation, expression in advanced pathological stages alongside distant metastasis, inhibition of apoptosis, and association with overall poor prognosis in multiple cancers [91–96]. While there are observed correlations between *HOTTIP* and vitamin D receptor signaling [97] as well as p21 silencing [91], the main role of *HOTTIP* described in cancer progression is its ability to utilize three-dimensional chromatin looping structures. These allow *HOTTIP* to regulate *cis*-members of the HOXA cluster by recruitment of WDR5 to drive H3K4me3 deposition into chromatin, activating target gene expression [98].

### The MEN (MALAT1-NEAT1) locus

The MEN locus is located on chromosome 11 at p13.1 and harbors both the *MALAT1* and *NEAT1* lncRNA genes. *NEAT1* is about 53kb upstream of the 5′ end of *MALAT1*, and both transcripts are deregulated in LUAD [99, 100].

#### MALAT1 (metastasis-associated lung adenocarcinoma transcript-1; multiple endocrine neoplasia-alpha)

This is a single exon gene originally identified as expressed specifically in lung cancer. Because it is associated with poor prognosis and distant metastasis in NSCLC [101–103], along with other cancers [102, 104–107], much of the emphasis in studying this gene is to utilize it as a prognostic biomarker [108, 109]. Identified over two decades ago, this functional RNA has been the sole subject of previous reviews [110, 111] and was initially implicated in RNA splicing through extensive studies *in vitro*.

Functionally, *MALAT1* interacts with Suz12 resulting in decreased expression of E-cadherin, a cell adhesion molecule essential in maintaining epithelial architecture. The loss of E-cadherin is a commonly observed phenomenon in cancers of epithelial origin, and co-occurs with upregulation of N-cadherin and fibronectin. This ultimately leads to metastasis, as reported in bladder cancer [112]. In addition, knockout studies of *MALAT1* have demonstrated that in lung cancer, *MALAT1* can also directly regulate the expression of pro-metastatic genes [99]. These observations tie together the poor prognosis and increased metastatic behaviors observed when *MALAT1* is over expressed in tumors.

#### NEAT1 (nuclear paraspeckle assembly transcript 1; Nuclear Enriched Abundant Transcript-1)

This is another single exon gene that is transcribed from the same locus as *MALAT1*. It exhibits many similar characteristics with *MALAT1*, including tumor recurrence [113], poor prognosis [114], and metastasis [115, 116]. Of special note, the close proximity of *NEAT1* to *MALAT1*, and their similar roles as oncogenes in multiple cancers suggests that the entire locus may be subject to aberrant regulation in cancer [117]. Indeed, several studies have demonstrated a correlation between *MALAT1* and *NEAT1* expression [115]. While *NEAT1* acts as part of chromatin remodeling complexes [118], less is understood about a direct functional role for *NEAT1* in carcinogenesis. *NEAT1* serves as a scaffold for nuclear paraspeckle formation [119–121], which accumulate in response to DNA-damaged induced genotoxic stress [122, 123]. It is possible that *NEAT1* acts to promote carcinogenesis directly by abrogating the stressors placed on the genomes of cancer cells. Further research is needed to determine if *NEAT1* plays a direct role in oncogenesis, or if instead the *MALAT1/NEAT1* locus is under mutual regulation, with *NEAT1* upregulation in cancer being a byproduct of its genomic proximity to *MALAT1*.

### Unclear mechanism(s) affecting cancer progression

Highlighting the need for further research, there are still several lncRNAs which are deregulated across cancer types, yet have not undergone in-depth functional characterization. This lack of mechanistic understanding hinders further investigation into the application of targeted therapeutics toward these deregulated lncRNAs, for concerns regarding off-target effects. One such transcript is *CCAT2*. It was originally identified as having LUAD-specific expression [124], but is now implicated in a host of cancers [125, 126] and associated with smoking [127]. It contains rs6983267, a single nucleotide polymorphism (SNP) identified through genome-wide association studies (GWAS) as conferring an increased risk of prostate and colorectal cancer [128–130]. This gene lies within the 8q24 ‘gene desert’ hotspot that is home to the MYC oncogene and is associated with numerous cancers [131–134], highlighting the significance of lncRNAs in genetic predisposition to cancer. However, rs6983267 has not been associated with LUAD risk in the numerous LUAD GWAS studies performed to date. Instead, it appears that copy number alterations in 8q24 occur frequently in lung cancers, suggesting an alternate mechanism other than SNP regulation of the *CCAT2* transcript in lung cancer pathogenesis. Mechanistically, *CCAT2* can alter cancer metabolism depending on the allele transcribed through altered binding affinity to pre-mRNA cleavage (CFIm) splicing factors [135]. However, little is known regarding how this altered splicing affects other cellular processes, or whether the differing alleles of *CCAT2* target the splicing complex to different chromatin locations to affect cancer development. This is but one example of the many lncRNAs that have been identified as deregulated in LUAD. Below, we highlight recent methods that have taken a more systematic approach to identifying the extent of lncRNA deregulation in LUAD, and what, if anything, is known about these genes.

## GENOME-WIDE ANALYSIS OF LNCRNAS IN LUAD

Original attempts to characterize the lncRNA landscape in LUAD were performed using microarray technology. While these arrays were designed to target mRNAs, many unaccounted-for exons that were later classified as lncRNAs were included in several platforms, notably the Affymetrix Human Exon 1.0 ST Array. Illumina-developed arrays contained less information on lncRNAs due to their design emphasis on 3′UTR targeting, however several lncRNAs were included under the ‘LOC_” definition. Using this probe-based approach several studies were able to identify lncRNA expression profiles in lung cancer and perform preliminary analysis [136–139], the results of which have been conveniently collated by lnc2cancer [140]. However, the discernable drawbacks of such techniques include the lack of discovery and low-expression levels of lncRNAs, thwarting detection efforts. With the advent and widespread adoption of RNA sequencing technology, the ability to detect novel transcripts had increased exponentially. Indeed, the rate at which the non-coding RNA transcriptome expanded has rapidly outpaced the identification of mRNA genes over the last five years.

Adding to the discovery landscape was The Cancer Genome Atlas (TCGA). LUAD samples from TCGA [15] underwent whole section RNA-seq analysis using Illumina TruSeq technology. This allows for polyA selection to minimize genomic contamination; however, it eliminates any non-polyadenyated signal from the final sequence alignments, therefore expression of only the poly adenylated lncRNAs were captured with this method. While the main purpose of RNA-seq analysis performed by TCGA was to quantify mRNA expression, several groups have utilized this dataset for dual interrogation of lncRNA transcriptome changes. LncRNAtor [141], MiTranscriptome [142], and The Atlas of Noncoding RNA In Cancer [TANRIC] [143] have each performed re-analysis of RNA-seq data from TCGA data to detect lncRNAs, with differing results based on the reference genomes utilized, filtering criteria, lncRNA references databases, and incorporation of secondary data sets. Their differing results highlight how alternate bioinformatic approaches can vastly affect the results of an analysis.

LncRNAtor showcases a re-analysis of several NGS datasets. They constructed a reference lncRNA library that included sequence from the EMSEMBL, lncRNAdb, HGNC and MBI datasets. They then analyzed each transcript for phylogenetic conservation and filtered transcripts against protein coding potential to arrive at a consensus reference set. Against this pipeline they re-aligned over 200 large-scale NGS datasets from 23 different cancers, including from GEO, ENCODE, and TCGA. Of these, only TCGA LUAD and LUSC dataset were specific for lung cancers. Their reanalysis identified 860 lncRNAs significantly differentially expressed between LUAD tumors and non-paired normal lung (adjusted p-value <0.01). However, many of the transcripts annotated as lncRNAs included known protein coding genes, such as *MMP12* and *UHRF1*, suggesting that inadequate filtering for coding potential may have inadvertently included protein coding genes. By comparison, lncRNAtor computed that 15,331 mRNAs were differentially expressed between LUAD tumors and normal tissue. The vast difference in the magnitude of changes (10-fold greater number of differentially expressed mRNAs) indicates that their analysis showed more variability in mRNAs then in lncRNAs. However, follow up work will be needed to determine if this is a reproducible phenomenon across cancers and sample sets.

MiTranscriptome was a re-analysis of data generated by TCGA, the Michigan Center for Translational Pathology, and ENCODE, developed to discover novel lncRNAs involved in cancer. Utilizing these sample sets, 58,674 lncRNA genes were identified, 1,150 of which were differentially expressed in LUAD (and obtainable through their portal linked to the UCSC genome browser). While their dataset has been made accessible via the BETA portal, as of July 2016 it does not contain the entire statistical analysis presented in their paper.

TANRIC contains the systematic re-analysis of TCGA data from over 20 cancer types, one of which was LUAD. Included in their analysis were 488 LUAD tumors and 58 unmatched normal lung sections from TCGA, along with re-analysis of RNA-seq data derived from the SEO study [16] of Korean LUAD cancer patients (83 LUAD and 77 matched normal samples). However, TANRIC focused on tumor heterogeneity and correlation with clinical covariants rather than simple tumor/normal comparisons, which can be influenced by issues such as tumor purity, degree of necrosis and other confounders.

**Figure 3:**
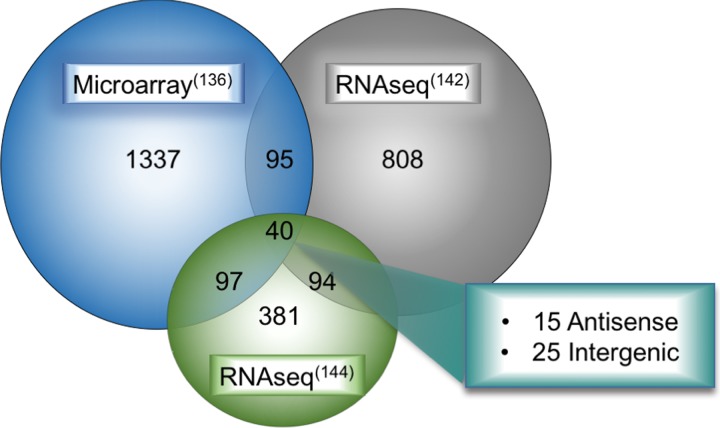
Overlap of deregulated lncRNAs in LUAD between multiple large-scale bioinformatic studies Results from microarray analysis, *de novo* RNA-seq transcriptome assembly of TCGA LUAD datasets from the Maher study on LCALs (supplementary data file 5 from their study which includes LUAD-specific lncRNAs), and robust statistical analysis of multiple lung cancer datasets were overlapped in the hg19 UCSC genome browser to determine a unifying set of lncRNAs deregulated in all three studies. These forty deregulated lncRNAs fall broadly into two categories, 15 were antisense transcripts and the remaining 25 were intergenic genes.

Another study by the Mather group took this analysis a step further [144]. After initially performing tumor-normal differential analysis and finding 592 altered lncRNAs, they then subtracted differentially expressed lncRNAs in multiple cancers to arrive at a lung cancer specific deregulated lncRNA class (including LUAD and LUSC). Their analysis also included re-analysis of the SEO dataset and a smaller subset of TCGA LUAD samples as performed by TANRIC (55 LUAD tumors and matched normals, with another 243 unmatched tumors). For the purposes of this review, the differentially expressed lncRNAs released by miTranscriptome, the Mather group, and microarray data [136] were compared (Figure 3). In this way, we referenced the newest technologies as well as compared them to the older microarray data, to provide a more refined review of the current state of LUAD-associated lncRNA discovery. Forty transcripts were differentially expressed between tumor and normal in all three data sets. We have segregated these forty differentially-expressed lncRNAs into stand-alone genes, heretofore labeled as intergenic lncRNAs (Table 2), and those that occur antisense to a protein-coding gene (Table 3). We have highlighted for further discussion several of these with a high degree of validation and prior mechanistic work, but all are potential candidate driver genes for LUAD.

**Figure 4:**
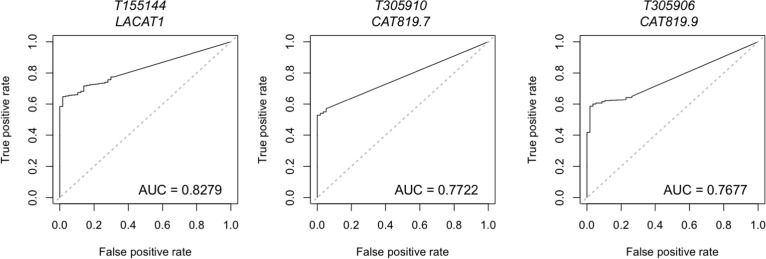
Lineage-specific lncRNAs identified in LUAD by miTranscriptome Data on lineage and cancer-specific LUAD lncRNAs was obtained from miTranscriptome. The top three lncRNAs with specificity to LUAD are shown. AUC = Area under the curve. False positive rate and false negative rate were generated using miTranscriptome-calculated expression levels for primary LUAD and normal lung tissue samples generated by TCGA. ROC curves were generated using the ROCR package in R.

**Table 2:** Differentially expressed intergenic lncRNAs

Level of Validation	Genecode ID	Alternate ID	Position (GRCh37/hg19)
Known Genes *tsl1*	*LINC00880*	NR_034007	chr3: 156799455-156840793
	*MIR3945HG*	TCONS_00008359	chr4: 185748077-185776905
	*LUCAT1*	TCONS_00010402	chr5: 90597873-90621000
	*PVT1*	ENST00000504719	chr8: 128806778-129113503
	*SFTA1P*	TCONS_00018434	chr10: 10825866-10837007
	*LINC00460*	ENST00000439790	chr13: 107028910-107030941
	*LINC00197*	TCONS_00023799	chr15: 95752084-96051089
	*MSN*	TCONS_00016990	*chrX*: 64808260-64845760
Validated Genes *tsl2-tsl3*	*MIR4435-2HG*	TCONS_l2_00015916	chr2: 112186885-112268567
	*CTB-43E15.1*	ENST00000523242	chr5: 173069521-173085197
	*HCG15*	TCONS_l2_00024132	chr6: 28953517-28959134
	*LINC00968*	ENST00000522511	chr8: 57401656-57472382
	*LINC01290*	ENST00000566787	chr16: 10608698-10622059
	*LINC00511*	ENST00000579631	chr17: 70319263-70599647
	*RP11-353N14.2*	TCONS_l2_00011006	chr17: 77796264-77801616
	*LINC00665*	ENST00000586345	chr19: 36795481-36822667
	*LINC00478*	ENST00000428669	chr21: 17553910-18013444
Predicted Genes *tsl4-tsl5*	*RP11-815M8.1*	TCONS_00000738	chr1: 222054322-222158306
	*LINC00152*	ENST00000331944	chr2: 87754947-87821037
	*RP11-83M16.6*	ENST00000510621	chr5: 66995256-67198428
	*RP11-359M6.1*	ENST00000548359	chr12: 79933982-79944315
	*RP11-1008C21.2*	TCONS_00023630	chr15: 38360990-38365188
Predicted Genes *tslNA*		TCONS_00007953	chr4: 1546984-1555291
		TCONS_00025436	chr17: 53690342-53725799
		TCONS_00029745	chr22: 50981205-50983413

LncRNAs that were deregulated in three studies (from Figure 3) that also occupy chromosomal regions in between mRNAs. LncRNAs were segregated by their Gencode transcript confidence level (tsl1 = highest quality, full transcript is validated; tsl2-3, one or many spliced ESTs are validated; tsl4-5 = one or none ESTs support the validity of the transcript, and those ESTs are suspect.) Those without a tsl ranking do not have a representative transcript in Gencode. All coordinates span the entire transcript length and are hg19 genome-based. The *MSN* transcript overlaps in the same orientation the *MSN* mRNA, therefore there is a high probability that this is not truly a lncRNA (and thus is greyed out in figure).

**Table 3:** Differentially expressed anti-sense lncRNAs

*Genecode*	Alternate IDs	Anti-Sense Gene	Multi-exonic	Position (GRCh37/hg19)
*LOC101928370*	RP4-575N6.1	*S1PR1*	YES	chr1: 101701238-101702084
*LINC00883*	DUBR	*LINC00882*	YES	chr3: 106959538-107045811
*LINC00312*	LINC00312	*LMCD1*	NO	chr3: 8613467-8634810
*LHFPL3-AS2*	RP11-325F22.5	*LHFPL3*	YES	chr7: 104558006-104567077
FEZF1-AS1	FEZF1-AS1	*FEZF1*	YES	chr7: 121945003-121945871
*HSPC324*	RP11-251M1.1	*EGFL7*	YES	chr9: 139541826-139554873
*LOC105369340*	RP11-783K16.5	*PPP1R14B*	YES	chr11: 64014525-64015649
*LOC101929340*	RP11-677M14.3	*ESAM*	YES	chr11: 124632326-124635257
SBK1-AS1	RP11-57A19.2	*SBK1*	YES	chr16: 28270020-28303385
FENDRR	FENDRR	*FOXF1*	YES	chr16: 86508050-86542705
*TBX2-AS1*	RP11-332H18.5	*TBX2*	YES	chr17: 59470732-59477096
	RP11-720L2.4	*COLEC2*	YES	chr18: 314886-319165
GATA6-AS1	GATA6-AS1	*GATA6*	YES	chr18: 19746858-19748929
*LINC01271*	RP11-290F20.2	*LINC01270*	YES	chr20: 48909256-48937879
*LINC00649*	LINC00649	*ATP5O*	YES	chr21: 35295736-35351160

The Gencode annotation for each lncRNA is indicated, along with the genomic coordinates of the lncRNA and the mRNA that is transcribed in the antisense orientation. All but *LINC00312* are multi-exonic, indicative of splicing. The hg19-based lncRNA coordinates are listed.

## INTERGENIC DISCOVERY-BASED LNCRNAS AND THEIR FUNCTIONAL IMPLICATIONS

Intergenic lncRNAs are herein defined as those lncRNA genes located in the space between protein coding genes. Prior evidence has shown that lncRNAs occurring *in cis* with protein coding genes can loop back and affect the nearby mRNA [98]. However, for intergenic lncRNA genes, their distal location makes functional predictions difficult. Instead, intergenic lncRNA can mediate their function *in trans* through a variety of mechanisms, such as their involvement with chromatin remodeling complexes [145]. Here, we focus on four lncRNAs that came from our review of LUAD transcriptomic profiling (Table 2).

### LUCAT1: (lung cancer associated transcript-1, also known as smoke and cancer-associated lncRNA-1, SCAL1)

is a multi-exonic lncRNA located deep within the gene desert of chromosome 5q14. This lncRNA is upregulated by cigarette smoke *in vivo* and *in vitro* through activation of the NRF-2 transcription factor [146]. NRF-2 (also known as NFE2L2) protects cells from oxidative stress and cigarette smoke toxicity [147], but its overexpression in LUAD cell lines results in drug resistance [148]. Consistent with dichotomous nature of the NRF-2 response [149], downregulation of *LUCAT1* also results in smoke-mediated cell death [146] suggesting *LUCAT1* may in part mediate the response of NRF-2 to oxidative stress. In addition, *LUCAT1* is upregulated in cisplatin-resistant ovarian cancer [150]. It remains unclear whether *LUCAT1* targets novel downstream genes involved in oxidative stress or whether it aids NRF-2 in activating NRF-2-dependent genes. Further mechanistic research on *LUCAT1* can elucidate if the oxidative stress response is related to or independent from *LUCAT1* upregulation by chemotherapy.

### PVT1 (plasmacytoma variant transcript 1)

is transcribed ∼60kb downstream of the MYC oncogene, and both reside within the 8q24 locus which undergoes copy number amplification in several cancers [151, 152]. Although MYC is an established oncogene, *PVT1* is also emerging as a prominent player in cancer. A recent study illustrated that some MYC-driven cancers are dependent on *PVT1* activity, as *PVT1* could stabilize MYC protein levels by preventing MYC phosphorylation [153]. In addition, silencing of *PVT1* in *PVT1*/MYC amplified cancers resulted in apoptosis, whereas MYC silencing had no effect, implying *PVT1* has a MYC-independent role in blocking apoptosis. The inhibition of apoptosis due to *PVT1* overexpression may be partially due to its role in silencing the *LATS2* gene via recruitment of EZH2 to its locus, inducing chromatin remodeling and gene silencing [154]. The LATS2 tumor suppressor is involved in a variety of functions, including induction of apoptosis and cell cycle control [155, 156]. Inhibition of LATS2 was previously observed in NSCLC, and *PVT1* overexpression was found to correlate with poorer overall prognosis [154].

The *PVT1* locus also contains multiple miRNA genes, including miR-1204, miR-1205, miR-1206, miR-1207-3p, miR-1207-5p, and miR-1208 [157]. Of interest, both miR-1204 and miR-1207-5p have demonstrated tumor suppressive properties [158, 159]. Surprisingly, p53 mediates the transcriptional expression of both *PVT1* and miR-1204 [158]. In addition, ectopic expression of miR-1204 induced p53-mediated growth inhibition in HCT116 cells. Therefore, induction of transcripts from the 8q24 locus results in lncRNAs that promote oncogenesis, and paradoxically, miRNAs that inhibit tumor promotion via p53. This may seem contradictory, but there have been reports of p53 mediating pro-survival pathways during DNA repair [160], such as p53 activation of p21/NRF2 signaling [161]. How this tight balance between pro-survival during DNA repair and apoptosis/cell death is disrupted in cancer will require further research in the downstream targets of the *PVT1* locus transcripts. The significance of other miRNAs inhabiting the 8q24 locus in p53-mediated signaling is unknown at the time of this writing.

Targeted therapy against 8q24 amplified cancers has remained challenging due to MYC being essential and in high abundance across normal tissue [162]. Because *PVT1* is less abundant in across normal tissue and possesses a protective role for MYC protein, *PVT1* appears to be a promising target for 8q24 amplified cancers.

### SFTA1P (surfactant associated 1 pseudogene)

Surfactant signaling is the distinguishing hallmark of alveolar epithelial type 2 (AT2) cells, a purported cell of origin for LUAD [163]. *SFTA1P* expression is correlated with other components of the surfactant machinery [164], and elevated *SFTA1P* levels indicate a better prognostic outcome for LUAD cancer patients (cox p-value = 0.009)[143]. This indicates that *SFTA1P* may hold potential as a biomarker of outcome prediction. However, because this gene is co-expressed with markers of differentiated AT2 cells, the loss of *SFTA1P* in a subset of LUAD cancers may be reflective of the overall differentiation state of the tumors. Moreover, the *SFTA1P* pseudogene is not located within genomic proximity to any of the other surfactant-protein producing genes, and the mechanisms (if any) by which *SFTA1P* functions remains unknown.

### LINC00460

This lncRNA is a multi-exonic, intergenic lncRNA over 100kb from the nearest mRNA gene, *EFNB2*. In addition to being found overexpressed in LUAD in the above studies, *LINC00460* is upregulated in head and neck squamous cell carcinoma, kidney carcinoma, and pancreatic cancer [165, 166]. While expression of *LINC00460* is correlated with *EFNB2* in LUAD (R=0.54, p=4.35e-28(143)), little research into the function or application of this lncRNA has been performed. The neighboring gene, *EFNB2*, encodes for EphrinB, one of many ligands for the Ephrin tyrosine kinase receptor. Much has been done implicating EFNB2 and the EphrinB receptor in development and progression of lung cancer [167–169]. However, the role *LINC00460* plays in this process, if any, has yet to be determined.

## LUAD ANTISENSE TRANSCRIPTS AND THEIR RELATION TO NEARBY PROTEIN CODING GENES

Antisense transcription has been observed at the transcription start site of numerous protein coding genes [170, 171]. This class of antisense transcripts range from siRNA [172] to antisense lncRNAs, such as *HOTAIR* [173]. Many have documented antagonistic activity, from epigenetic regulation [174] to direct disruption of the transcriptional machinery [175]. Here, we highlight a few antisense lncRNAs identified through bioinformatics analysis to be involved in LUAD, while the entire list is summarized in Table 3.

### FENDRR (FoxF1 adjacent non-coding developmental RNA)

This gene is transcribed in the antisense direction from the adjacent FOXF1 transcription factor. As expected, expression of *FENDRR* is highly correlated to *FOXF1* (R=0.816, p value =1.52e-85 [143]). The FOXF1 transcription factor is implicated in mesoderm development, and similarly *FENDRR* is implicated in embryogenic mesoderm formation, specifically heart development [176, 177]. FOXF1 is overexpressed in LUAD and plays a central role in regulating epithelial-to-mesenchymal transition by promoting tumorigenesis of adenomas toward adenocarcinomas [178, 179]. Mechanistically, *FENDRR* is proposed to affect the extracellular matrix due to its inverse correlation with fibronectin1 expression in gastric cancer cell lines [180]. Disrupting fibronectin1 is associated with tumor migration and metastasis [181]. Adding extra weight to the argument that FENDRR may promote EMT and metastasis, Xu et al., found that lower *FENDRR* expression correlates with higher metastatic potential and poorer outcomes in LUAD patients [180]. Similar to *HOTAIR* and *MEG3*, *FENDRR* appears to form RNA:DNA triplexes to recruit PRC2 complex during embryonic mesoderm patterning, which when disrupted leads to deformation of the heart and embryo death [182].

### FEZF1-AS1 (FEZ family zinc finger 1- antisense 1)

FEZF1 (also known as ZNF312b) is a zinc finger transcriptional repressor that is an epigenetically-regulated oncogene in gastric cancer [183]. This protein promotes proliferation via kRAS-oncogene activation [184]. *FEZF1-AS1* positively regulates expression of FEZF1 mRNA expression *in vitro* as well as in the TANRIC analysis of TCGA LUAD data (p=2.85e-104). *FEZF1-AS1* is upregulated in human primary colorectal carcinoma, and affects colorectal cancer cell proliferation, metastasis, and invasion [185]. However, it remains to be determined how *FEZF1-AS1* and *FEZF1* interact mechanistically.

### SBK1-AS1 (SH3 domain binding kinase 1 antisense-1; RP11-57A19.2)

SBK1 is a serine/threonine kinase family member implicated in ovarian serous adenocarcinoma cell survival [186]. Several serine threonine kinase family members exhibit oncogenic behavior, such as PIM1 and BRAF. These are attractive therapeutic targets, as small molecule inhibitors have proven effective in halting cancer progression [187, 188]. *RP11-57A19.2* is transcribed in an antisense direction from the *SBK1* promoter, and the two have correlated expression in multiple cancer types (TCGA-LUAD R=0.597, TCGA-OVR=0.701, TCGA-BRCA=0.671, p=3.95e-108 [143]), however little to nothing is known about the expression, function, and regulation of *RP11-57A19.2*.

### GATA6-AS1 (GATA-binding protein-6 antisense-1)

GATA6 is an important regulatory transcription factor in alveolar epithelial cell biology [189, 190]. The antisense *GATA6-AS1* is correlated to GATA6 expression during lung development, albeit with more cell-type specific restriction than the GATA6 transcription factor [191], as well as in TCGA LUAD datasets (R=0.772, p=3.53e-71 [143]). Overexpression of *BM742401*, an expressed sequence tag which corresponds to *GATA6-AS1*, reduced cancer metastasis and decreased secretion levels of MMP9, though the mechanism by which *GATA6-AS1* mediated these effects remains unknown. Interestingly, these authors investigated whether *GATA6-AS1* overexpression affected the expression of GATA6, but found no change (this data not reported) [192]. Expression of GATA6 in LUAD is associated with a more differentiated state, and reflective of that, with better overall patient survival [193]. Whether the expression of *GATA6-AS1* functions to maintain the differentiated state, or is merely a passive reflection of differentiation, remains to be determined.

## CLINICAL RAMIFICATIONS

Lung cancer remains the leading cause of cancer-related death in the United States. Although improvements in surgical treatment and chemotherapies have shown some progress, the 5-year survival rate lingers at ∼15% [12]. In addition, NSCLC is composed of several differing subtypes, each with their own set of heterogenic factors that result in cancer. The array of molecular mechanisms implicated in the genesis of this disease underscores the need more accurate prognostic markers, to implement therapies targeted to the specific pathways disrupted in each disease subtype and inform clinicians on predicted patient outcomes.

Non-coding RNAs hold promise as biomarkers for a variety of cancers. For instance, increased miR-486 levels showed efficacy as a blood-based biomarker for early detection of NSCLC, and lower levels of miR-486 post-surgical resection was an effective predictor of recurrence-free survival of NSCLC patients [194]. As mentioned above, *MALAT1* and *LUCAT1* also hold promise as prognostic biomarkers as their elevated expression is linked with poorer overall survival [195]. However, enthusiasm surrounding *MALAT1*’s applicability to early detection was tempered by research describing its overall sensitivity of detection at only 56%, meaning almost half of true positive LUAD cases would be missed using this biomarker alone [196]. To circumvent this issue, *MALAT1* was included in a panel of lncRNAs to improve the detection sensitivity while maintaining the specificity for LUAD. The other lncRNAs included in the panel were: *ENST00000540136*, *NR034174*, *uc001gzl.3*, *uc004bbl.1*, and *ENST00000434223*. When combined, this panel outperformed any individual lncRNA in the training set. The testing set reached an AUC of 0.978 for tumor identification, with 92% sensitivity and 98% specificity [138], reinforcing the notion that the combining the inherent cell-type specificity of many lncRNAs with the sensitivity of others can aide in the development of early detection tools.

Separate from this panel of biomarkers, miTranscriptome has also undertaken the task of calculating the specificity of expression within each individual cancer for the entire lncRNA transcriptome. They identified 25 lncRNAs that demonstrated statistically robust specificity for LUAD [142], only five of which were previously annotated. These genes included *PVT1* (mentioned above as adjacent to the *MYC* oncogene), *DYPD-AS1, LACAT24,* and *LACAT8* (Table 4). It is interesting to note that this analysis included 23 cancerous tissues alongside 12 normal tissues, a step not typically undertaken when assessing the feasibility of early detection tools. As no single study can yet address the wide diversity of cell types present in the human body [197], including as many cell types as is available can nevertheless decrease the risk of investing heavily in the development of a biomarker, only to see it fail in clinical trials due to off-target effects.

**Table 4:** miTranscriptome-defined LUAD lineage-specific lncRNAs

miTranscriptome	Alternate IDs	Position (GRCh37/hg19)	Genomic Context	Patient Outcome
*CAT1100.3*	*PVT1*	chr8:128996355-129130070 (−)	*Adjacent to MYC oncogene*	*Correlated to MYC expression and tumor progression. PVT1 presence indicates poorer prognosis for patients*
*DPYD-AS1.2*	*NR_046590.1*	chr1:97720954-97751573 (+)	*Antisense to DYPD*	*DYPD implicated in increased toxicity to patients treated with 5FU.*
*LACAT24*	*LOC101927132*	chr16:47936832-47961855 (+)	*Intergenic*	*Unknown*
*LACAT8.1*	*LACAT8*	chr12:131471968-131478539 (−)	*Antisense to GPR133 (ADGRD1)*	*GPR133 upregulation correlated to poorer survival in GBM*

miTranscriptome-calcuated lncRNAs with lineage specificity to LUAD.

In the future, testing for lncRNA expression could also yield gains in personalized medicine. For instance, overexpression of *HOTAIR* in LUAD results in chemo-resistance towards cisplatin [198]. *HOTAIR*-induced drug resistance was attributed in part to downregulation of p21. Although the mechanism by which *HOTAIR* regulates p21 remains unknown, previous studies have reported EZH2 is involved in p21 suppression [199]. Considering the known involvement of *HOTAIR* with the PRC2/EZH2 complex, *HOTAIRs* role in mediating p21 suppression and cisplatin resistance might be due to epigenic modifications. Therefore, testing for *HOTAIR* expression after resection may insulate a subset of the patient population from having to undergo chemotherapy and instead direct them toward promising EZH2 inhibitors, such as E7438, currently under evaluation in clinical trials [200].

A lncRNA with oncogenic activity and expression restricted to a specific cancer would be an ideal therapeutic target. To that end, anti-RNA treatments are currently being developed to diversify the options available to clinicians. However, many obstacles have arisen, including a lack of efficient delivery methods, RNA degradation, and aberrant immune system activation [201]. In addition, some lncRNAs have high turnover and low transcriptional expression, making them difficult to target effectively. In such cases, knowledge of pathway involvement is needed to develop effective treatments that target downstream signaling molecules affected by aberrant lncRNA activity.

## CONCLUSIONS

Over the last decade, lncRNAs have been recognized as a diverse class of macromolecules in terms of function and mechanism. Many lncRNAs have demonstrated functional activity in a wide range of cancers. While the lncRNA transcriptome is far from complete, we now have an appreciation of their diversity due to cell-type specificity. This characteristic can aid in the development of early detection methods and targeted therapies for multiple cancer types. Alongside these immediate applications, understanding the mechanism(s) by which these transcripts are regulated will shed light on the etiology of cancer development, allowing clinicians to implement better treatment strategies and improve overall survival rates.

While research in this field is still in preliminary stages, multiple large-cohort patient studies such as TCGA, ENCODE, and SEO alongside previous microarray studies have delineated a host of lncRNAs with potential for novel therapeutic strategies. As such, they have provided framework for the interrogation of molecular mechanisms that lncRNAs utilize in LUAD initiation, promotion, and progression. While in this review we focused on lncRNAs validated by three different studies, it is by no means exhaustive. It remains to be seen if other case-controlled, multi-ethnic cohorts will unveil an entirely new set of lncRNAs with implicit utility in the diagnosis and treatment of LUAD. It will be interesting to follow what discoveries that work will yield in the development of novel therapeutics and early detection strategies in the years to come.
